# Epidemiology of coinfection with soil transmitted helminths and *Plasmodium falciparum* among school children in Bumula District in western Kenya

**DOI:** 10.1186/s13071-015-0891-5

**Published:** 2015-06-11

**Authors:** Stella Kepha, Fred Nuwaha, Birgit Nikolay, Paul Gichuki, Tansy Edwards, Elizabeth Allen, Sammy M. Njenga, Charles S. Mwandawiro, Simon J Brooker

**Affiliations:** College of Health Sciences, Makerere University, Kampala, Uganda; Eastern and Southern Africa Centre of International Parasite Control, Kenya Medical Research Institute (KEMRI), Nairobi, Kenya; London School of Hygiene & Tropical Medicine, London, UK; KEMRI-Wellcome Trust Research Programme, Nairobi, Kenya

**Keywords:** Soil transmitted helminths, Hookworm, *Ascaris lumbricoides*, *Plasmodium falciparum*, Coinfection

## Abstract

**Background:**

Many school children living in Africa are infected with plasmodia and helminth species and are consequently at risk of coinfection. However, the epidemiology of such coinfection and the implications of coinfection for children’s health remain poorly understood. This study describes the epidemiology of *Ascaris lumbricoides*-*Plasmodium* and hookworm-*Plasmodium* coinfection among school children living in western Kenya and investigates the associated risk factors.

**Methods:**

As part of a randomized trial, a baseline cross-sectional survey was conducted among school children aged 5–18 years in 23 schools in Bumula District. Single stool samples were collected to screen for helminth infections using the Kato-Katz technique and malaria parasitaemia was determined from a finger prick blood sample. Demographic and anthropometric data were also collected.

**Results:**

Overall, 46.4 % of the children were infected with *Plasmodium falciparum* while 27.6 % of the children were infected with at least one soil transmitted helminth (STH) species, with hookworm being the most common (16.8 %) followed by *A. lumbricoides* (15.3 %). Overall 14.3 % of the children had STH*-Plasmodium* coinfection, with hookworm-*Plasmodium* (9.0 %) coinfection being the most common. Geographical variation in the prevalence of coinfection occurred between schools. In multivariable logistic regression analysis, hookworm was positively associated with *P. falciparum* infection. In stratified analysis, hookworm infection was associated with increased odds of *P. falciparum* infection among both boys (*P* < 0.001) and girls (*P* = 0.01), whereas there was no association between *A. lumbricoides* and *P. falciparum*.

**Conclusion:**

These findings demonstrate STH infections are still prevalent, despite the ongoing national deworming programme in Kenya, and that malaria parasitaemia is widespread, such that coinfection occurs among a proportion of children. A subsequent trial will allow us to investigate the implications of coinfection for the risk of clinical malaria.

**Electronic supplementary material:**

The online version of this article (doi:10.1186/s13071-015-0891-5) contains supplementary material, which is available to authorized users.

## Background

School children living in sub-Saharan Africa are commonly infected with helminth and plasmodia species, with many children harbouring multiple infections. For example, studies in east and west Africa indicate that between 13.5 % and 60 % of school children are concurrently infected with *Plasmodium falciparum* and different soil-transmitted helminth species [1–11]. Helminths are known to invoke strong immune responses [12] and it has been suggested that they affect malaria-specific immune responses which may, in turn, alter the risks of malaria parasitaemia and clinical disease [12, 13]. The occurrence of coinfection will also influence the planning of integrated intervention strategies that simultaneously tackle helminth infections and malaria [14]. Despite the growing awareness of the issue of helminth-plasmodia coinfection, there are few detailed and comprehensive data on coinfection and putative risk factors. Previous work in Kenya has shown that coinfection is more common among boys, is less common with increasing age, and highest among children from the poorest households [3, 4]. Other work in Côte d’Ivoire confirms that boys and children from poorer households are at greater risk of coinfection with soil transmitted helminths and *Plasmodium* spp. [9, 11]. The risk of coinfection is also associated with access to sanitation and clean water, recent deworming, and living in urban settings [3, 9]. Studies have in addition highlighted the marked spatial heterogeneity in the distribution of helminth-coinfection at both local and regional scales [3, 4, 8]. In this paper we describe results from school surveys carried out in Bumula District, western Kenya, conducted as part of screening surveys for an individually randomized trial investigating the impact of intensive anthelminthic treatment versus annual treatment on the risk of clinical malaria among school children in Bumula District, western Kenya (ClinicalTrials.gov NCT01658774). The aim of the present analysis is to describe the patterns of Ascaris-*Plasmodium* and hookworm-*Plasmodium* coinfection and investigate whether helminth species infection are associated with *Plasmodium* infection.

## Methods

### Study setting

The survey was conducted in Bumula District which is one of the sub-counties in Bungoma County, western Kenya, between February and June 2013. Bumula District is located at 1,320 m elevation and experiences an annual average rainfall of 2,428 mm, with the long rains occurring from March-May and short rains from October-December. Average annual minimum and maximum temperatures are 11 °C and 24 °C, respectively. The population of the area consists of indigenous Bukusu, a subtribe of the Luhya community. The economy is primarily rural subsistence agriculture, with some families growing sugar cane as a cash crop. The population is serviced by Bumula Sub-District Hospital, which serves about 180,000 people and a catchment area of approximately 250 km^2^.

The area was chosen because it experiences a high rate of malaria transmission and some of the highest prevalences of STH in Kenya [15, 16]. Malaria transmission is intense and perennial, with two seasonal peaks (April-June and November-December), and most malaria is caused by *P. falciparum*. Recent survey data indicated the prevalence of *P. falciparum* as 21.6 % [16]. Historically, helminth infections were highly prevalent in the area [17], but improvements in socioeconomic status and access to water and sanitation has reduced infection levels [18]. A national school-based deworming programme launched in 2009 and more recent data indicate that 25.1 % of school children are estimated to be infected with *Ascaris lumbricoides* and/or hookworm [4, 15]. As part of the national school deworming programme launched in 2012, school children were treated with 400 mg of albendazole in June 2012 and June 2013.

### Selection of schools and children

Representative schools of the 90 public primary schools in Bumula District were purposively selected for initial screening with the assistance of the district education and health officials. Initially, 30 schools were screened in January 2013 to identify those schools with highest prevalence of STH infection. Seven schools were excluded because of a low prevalence of STH, with 23 schools recruited into the study. The present study recruited children regardless of infection status, whereas the treatment trial will, in the first instance, recruit only children found to be infected with STH species.

### Survey procedures

Parent/guardians of children in class 1–6 were invited to attend sensitization meetings held at the selected schools. The study procedures were explained in a language with which they felt most comfortable, written informed consent was obtained from all parents who were willing to have their children be part of the study before any investigations were done.

Children were asked to provide stool samples which were examined in duplicate for presence of STH eggs by two different technicians using the Kato-Katz method. For the investigation of *P. falciparum* infections, finger prick blood samples were collected. Thick and thin blood smears were stained with 3 % Giemsa for 45 min and examined by microscopy. Parasite densities were determined from thick blood smears by counting the number of asexual parasites per 200 white blood cells, assuming a white blood cell count of 8,000/μl. A smear was considered negative after reviewing 100 high-powered fields. Thin blood smears were reviewed for species identification. All stool and blood slides were read by two independent microscopists; any discrepancies were resolved by a third microscopist. Ten percent of all samples were re-examined by a senior technician and discrepant (positive versus negative) slides were re-read by a third technician until a consensus was reached. Auxiliary temperature was measured using a digital thermometer and a malaria rapid diagnostic test (SD; Bioline) was performed for all children with fever (auxiliary temperature of <37.5 °C or with a reported history of fever in the previous 24 h). Any participant tested positive for malaria was treated with Coartem (Novartis; 20 mg artemether/120 lumefantrine) in accordance with the national treatment guidelines. Weight was measured to the nearest 0.1 kg using an electronic balance and height was measured to the nearest 0.1 cm using a portable fixed base stadiometer.

### Statistical analysis

Anthropometric indices, z-scores of height-for-age (HAZ), weight-for age (WAZ) and body mass index for age (BMIZ) were calculated using the AnthroPlus software for children aged 5–19 years [19]. Age was self-reported and because there were doubts over its precision a mid-year age was assumed. Weight-for-age was calculated only for the children aged 5–10 years. Children were classified as stunted, underweight or thin if their HAZ, WAZ and BMIZ were below −2 standard deviations from the reference medium [19].

The prevalence of each STH species together with the 95 % confidence interval (95 % CI) were calculated using binomial regression analysis taking into account clustering by school. Egg counts of duplicate slide readings were averaged and multiplied by factor 24 to obtain the intensity of infection expressed as eggs per gram (epg) of faeces. To allow for the over dispersed distribution of egg counts arithmetic mean epgs with their 95 % CIs were estimated using negative binomial regression model taking into account school clustering. Infection intensities were also classified into light and moderate to heavy infections according to WHO guidelines [20].

For purposes of this analysis, age was considered as categorical variable (5–8, 9–10, and 11–12 and <14 years), based on observed distribution. To investigate risk factors for *P. falciparum* infection, mixed effects logistic regression models were fitted taking into account school clustering. First, associations with sex, age (as categorical variable), and nutritional status (classified as underweight, thin or stunted) were investigated in univariable analysis. Explanatory variables significant at (*P* < 0.20) (based on likelihood ratio test) were considered for the multivariable logistic regression model. Backward-stepwise elimination was used to generate a minimum adequate model, excluded variables (*P* > 0.05) were retested in the minimum model. Associations between helminth species and *Plasmodium* infection were investigated using stratified analysis following the Mantel-Haenszel approach adjusting for the potential confounding of sex, age and presence of other helminth species. Overall odds ratios and odds ratios stratified by age and sex, their 95 % confidence intervals (CI) and associated P-value for Mantel-Haenszel ***χ***^**2**^ test are reported. Sensitivity analysis was done to compare children without complete data to those with complete data. Statistical analysis was performed using STATA version 12.0 (Stata cooperation, College Station Tx, USA).

### Ethical consideration

Ethical approval was provided by the Kenya Medical Research Institute and National Ethics Review Committee (SSC No. 2242), the London School of Hygiene &Tropical Medicine (LSHTM) Ethics Committee (6210), the Makerere School of Public Health, Institutional Review Board (IRB00005876). Prior to the study, school meetings were held during which the purpose and procedures of the study were discussed, parents had the opportunity to ask question, and willing parents were asked to provide written consent for their children to participate.

## Results

**Fig. 1:**
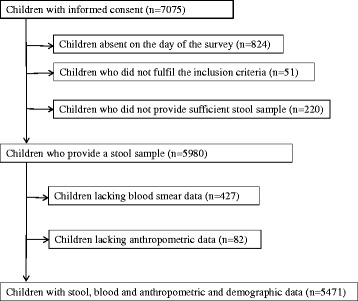
Study flow diagram showing compliance in a cross sectional survey conducted in school children in 23 schools in Bumula District, western Kenya

The 23 schools had an estimated total population of 8,800 of which informed consent was obtained for 7,075 children (80 %). Of these children, 51 did not fulfil the inclusion criteria, 824 were absent on the day of the survey, and 220 children did not either provide a stool sample or an adequate quantity of stool (Fig. 1). Overall, 509 children had missing data on malaria parasitaemia or anthropometric measures, and sensitivity analysis found that children with missing data were more likely to be underweight and thin and infected with *P. falciparum*, but less likely to be infected with helminth species (Additional file 1: Table S1). The characteristics of included children (*n* = 5,471) and the prevalence of parasitic infections are shown in Table 1. The mean age of children was 10.5 years (SD, 2.3) and 50.8 % were male. In terms of nutrition status, 3.4 % of children were classified as stunted, 23.9 % of children were underweight and 10.6 % were classified as thin. Underweight and thinness was more common in male than female children (28.9 % vs. 18.7 %, *P* < 0.001 and 12.8 % vs. 8.6 %, *P* < 0.001).

**Table 1:** Description of study participants, overall, and by sex. SD = standard deviation, CI = confidence intervals

Characteristic	Overall (n = 5,471)	Boys (n = 2,782)	Girls (n = 2,689)	*p*-value
Mean age (years, SD)	10.5 (2.3)	10.6 (2.6)	10.3 (2.4)	<0.001
Age-group (years, n (%))				
5-8	1294 (23.7)	624 (22.4)	670 (24.9)	
9-10	1413 (25.8)	677 (24.3)	736 (27.4)	0.263
11-12	1411 (25.8)	728 (26.2)	683 (25.4)	
13-18	1353 (24.7)	753 (27.1)	600 (22.3)	
Class (n, (%))				
Lower primary (1–3)	2711 (49.5)	1422 (52.5)	1289 (47.5)	
Upper primary (4–6)	2760 (50.5)	1360 (49.3)	1400 (50.7)	0.020
Stunted (n, (%))	186 (3.4)	106 (3.8)	80 (3.0)	0.080
Underweight (n, %)	1306 (23.9)	803 (28.9)	503 (18.7)	<0.001
Thinness (n %)	577 (10.6)	355 (12.8)	222 (8.6)	<0.001
Prevalence of helminth infection				
Hookworm (%, 95 % CI)	16.9 (14.6-19.5)	20.0 (17.1-23-4)	13.6 (11.5-16.0)	<0.001
*A. lumbricoides* (%, 95 % CI)	15.3 (12.2-19.2)	14.5 (11.6-18.3)	16.2 (12.7-20.7)	0.165
*T. trichiura* (%, 95 % CI)	0.3 (0.2-0.7)	0.30 (0.2-0.7)	0.5 (0.2-0.9)	0.444
Any STH (%, 95 % CI)	27.6 (24.8-30.8)	29.7 (26.4-33.4)	25.5 (22.3-29.1)	0.020
*S. mansoni* (%, 95 % CI)	0.2 (0.1-0.4)	2.8 (1.6-4.9)	1.6 (0.9-3.7)	0.100
Intensity of helminth Infection				
Hookworm (epg, 95 % CI)	41 (27–61)	54 (35–85)	27 (16–45)	0.008
*A. lumbridoides* (epg, 95 % CI)	758 (559–1028)	641 (455–901)	880 (647–1195)	0.005
*P. falciparum* infection (%, 95 % CI)	46.4 (41.7-51.6)	48.9 (44.1-54.1)	44.0 (39.2-49.3)	<0.001
Mean parasite density (parasites per μl blood)				
Uninfected (n, %)	2930 (53.7)	1423 (51.1)	1507 (56.0)	
Low (1–999) (n, %)	1576 (28.8)	836 (30.0)	740 (27.5)	0.319
Medium/high (≥1000) (n, %)	965 (18.6)	523 (18.8)	442 (16.4)	
Coinfection				
Hookworm-*A. lumbricoides* (%, 95 % CI)	4.7 (3.7-6.1)	5.00 (3.8-6.5)	4.5 (3.3-6.0)	0.448
Hookworm-*P. falciparum* (%, 95 % CI)	9.0 (7.4-11.0)	11.2 (9.3-13.6)	6.9 (5.5-8.7)	<0.001
*A. lumbricoides*-*P. falciparum* (%, 95 % CI)	7.8 (6.1-10.0)	7.80 (5.9-10.1)	7.8 (5.8-10.4)	0.904
Any STH-*P-falciparum* (%, 95 % CI)	14.3 (12.1-16.8)	15.9 (13.4-18.9)	12.5 (10.4-15.2)	0.002

### Single species infection

**Fig. 2:**
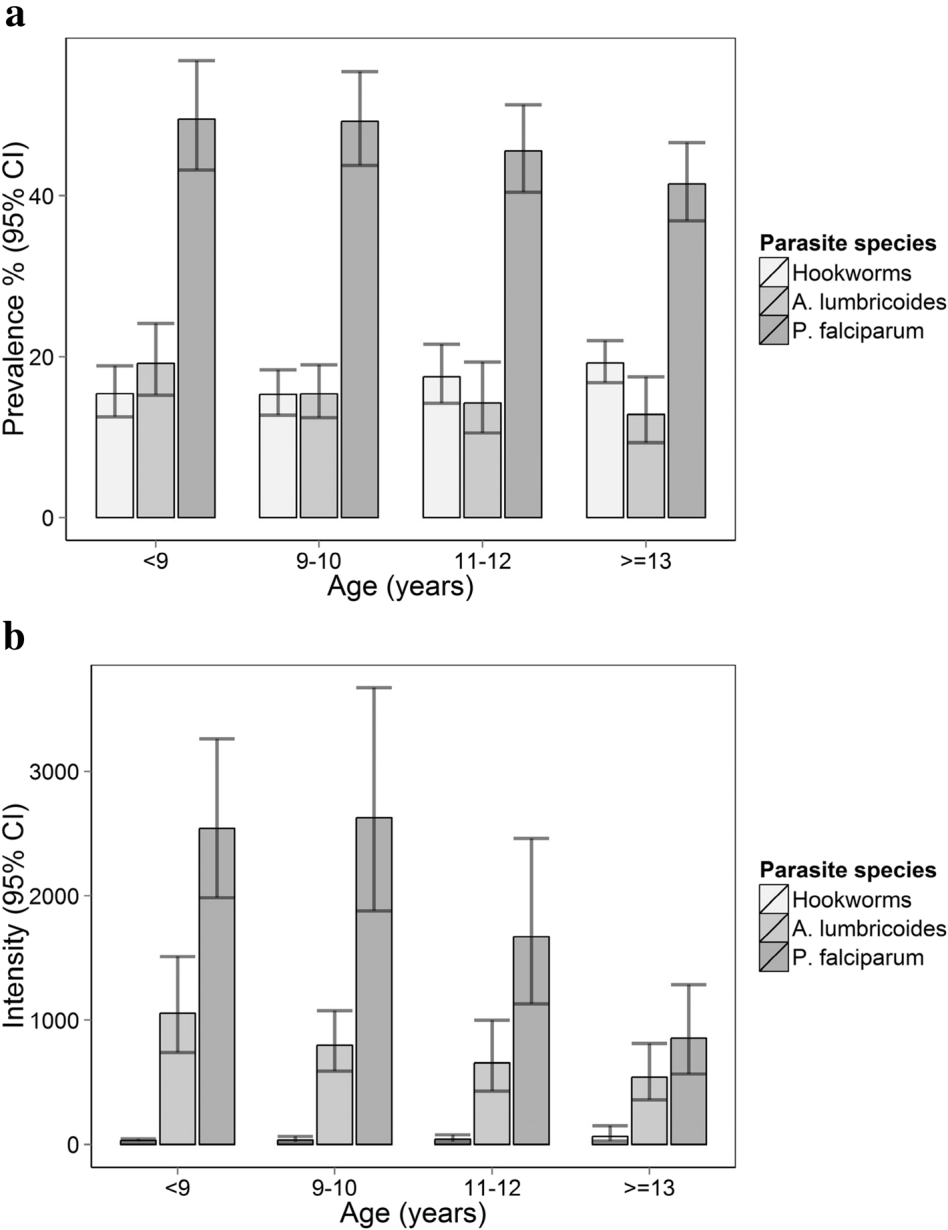
Prevalence (**a**) and intensity/density (**b**) of infections by age group among 5471 school children in 23 schools in Bumula District, western Kenya. 95 % confidence intervals were calculated based on binomial and negative binomial regression taking into account school clusters. Intensity was measured in eggs/g faeces for STH species and parasites/μl blood for Plasmodium falciparum

Overall, 27.6 % of the children were infected with any STH species. The most common species was hookworm (16.9 %), followed by *A. lumbricoides* (15.3 %); *T. trichiura* occurred rarely (0.3 %). Infections with multiple helminth species were relatively uncommon (4.7 %) (Table 1). Infection intensity was generally light as per WHO classification; 68 % of the *A. lumbricoides* and 97 % of the hookworm infections were light infections. Malaria was common: 46.4 % of children were infected with *P. falciparum* (the only species detected), with the majority of infections being light densities. Infection prevalence and intensity of hookworm and *P. falciparum* differed by sex*,* with boys being significantly more infected than girls, while *A. lumbricoides* and *T. trichiura* prevalence was comparable between the sexes. Age patterns of prevalence and intensity are shown by species in Fig. 2. The prevalence of *A. lumbricoides* was highest in children below the age of 9 years while the prevalence of hookworm peaked among children aged 13–18 years. The prevalence of *P. falciparum* decreased steadily with age (*P* < 0.001). For all the three species, intensity of infection was generally low and decreased with increasing age apart from hookworm.

### Coinfection

Overall, 14.3 % of children harboured STH-*Plasmodium* coinfection, with hookworm-*Plasmodium* coinfection being the most common combination (9.0 %). The prevalence of hookworm-*Plasmodium* coinfection was also significantly higher in boys than girls, but no sex difference was found in the prevalence of *A. lumbricoides*-*Plasmodium* coinfection. The prevalence of *A. lumbricoides-Plasmodium* coinfection was significantly different among age groups being common in the younger age group (5–8) (*P* = 0.001), hookworm-*Plasmodium* coinfection did not vary by age group *(P* = 0.613).

### Geographic distribution of single infections and coinfection

**Fig. 3:**
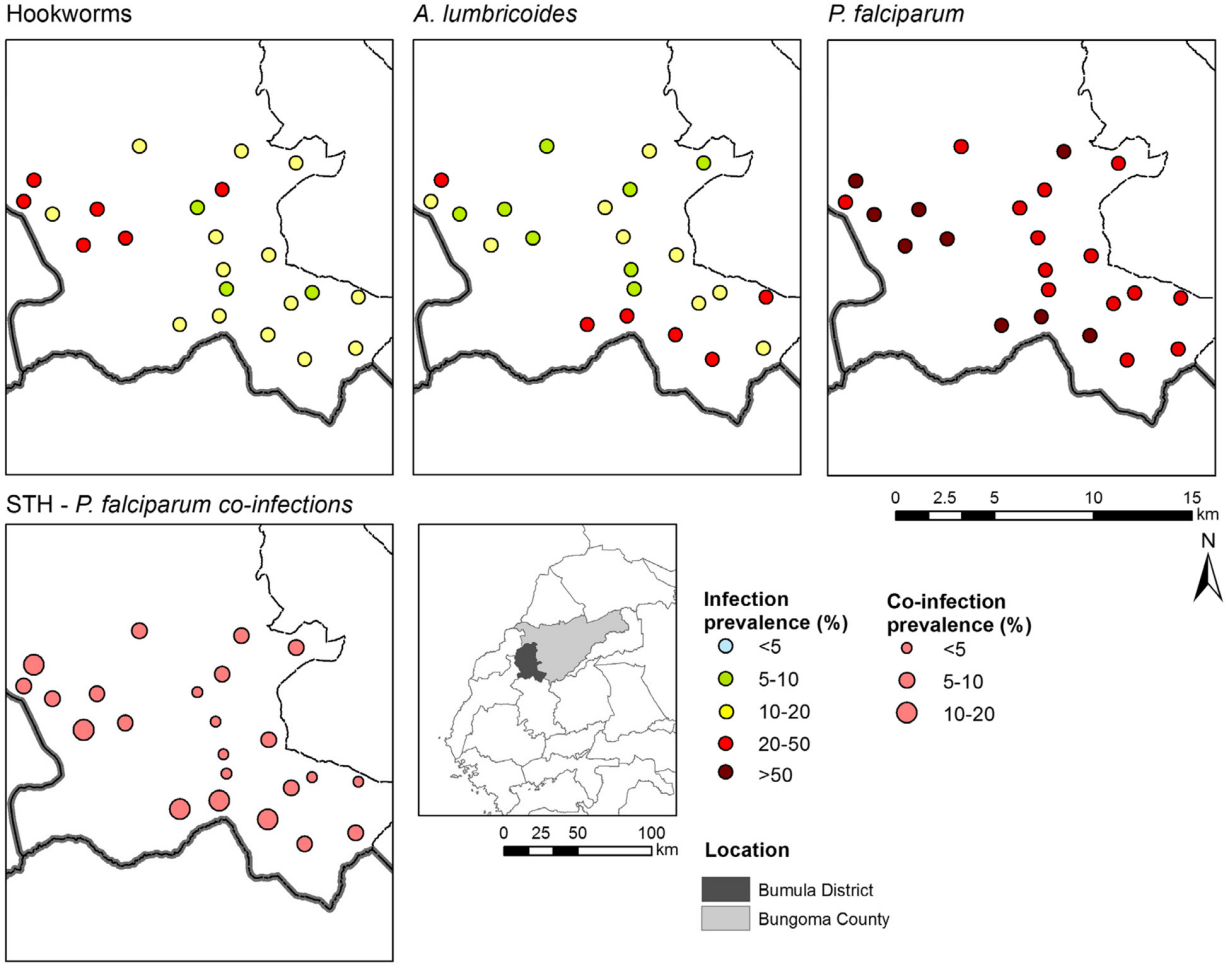
Geographic distribution of STH and Plasmodium falciparum infection and prevalence of STH-Plasmodium coinfections in 23 schools in Bumula District, western Kenya

The geographic distribution of single infection and coinfection by school is shown in Fig. 3. *P. falciparum* was common throughout the study area (range by school, 28.9-69.3 %), with highest prevalence in the western part of the area. The variation in the geographical distribution of STH was more pronounced and varied by species: hookworm was most common in the north-western part of the region (range by school, 7.9-30.0 %), while *A. lumbricoides* was more common in the eastern area (range by school, 5.8-34.4 %). Coinfection also exhibited geographical variation and coinfection was generally higher in schools with high hookworm or *A. lumbricoides* prevalence.

### Factors associated with *Plasmodium* infection

Table 2 presents the univariable and multivariable analysis of the factors associated with *P. falciparum* infection and shows that the odds of *P. falciparum* infection is associated with male sex, and age group. The analysis also provides strong evidence for ensuring association between *P. falciparum* and hookworm infection and modest evidence for an association between *P. falciparum* and *A. lumbricoides* infection. In order to further investigate the associations between helminth species and *P. falciparum* infections, we performed Mantel-Haenszel stratified analyses. Table 3 presents Mantel-Haenszel stratified (by age group and sex) odds ratios (OR) and indicates that, for boys, the observed association of hookworm and *P. falciparum* was age dependent, with significantly increased odds among children 5–8 years compared to other age groups. After adjusting for age group and *A. lumbricoides* infection, hookworm infection was associated with increased odds of *Plasmodium* infection among both boys (adjusted OR = 1.40, *P* = 0.0004) and girls (adjusted OR = 1.33, *P* = 0.01). Mantel-Haenszel stratified analysis for *A. lumbricoides* and *P. falciparum* association showed no association by age or sex (Table 4).

**Table 2:** Univariable and multivariable analysis for association between *Plasmodium falciparum* infection and potential risk factors among 5471 school children in 23 schools in Bumula District, western Kenya

Variable	Number with *P. falciparum*	Crude odds ratio (95 % CI)	*P*-value	Adjusted odds ratio (95 % CI)	*P*-value
Individual characteristics					
Sex					
Boys	1359 (48.9)	1			
Girls	1182 (44.0)	0.84 (0.75-0.94)	0.002	0.84 (0.75-0.94)	0.003
Age groups (years)					
5-8	641 (49.5)	1.44 (1.23-1.69)		1.51 (1.28-1.79)	
9-10	696 (49.3)	1.46 (1.25-1.70)	0.126	1.51 (0.29-1.78)	<0.001
11-12	643 (45.6)	1.23 (1.06-1.44)		1.25 (1.07-1.47)	
13-18	561 (41.5)	1		1	
Any STH infection	781 (51.7)	1.29 (1.14-1.46)	<0.001		
Hookworm infected					
Infected	428 (46.4)	1			
Uninfected	494 (53.6)	1.31 (1.13-1.52)	<0.001	1.27 (1.09-1.47)	0.002
Hookworm infection intensity					
Uninfected	2047 (45.0)	1			
Low	476 (53.5)	1.29 (1.12-1.49)			
Medium	18 (56.3)	0.83 (0.68-1.00)	0.710		
*A. lumbricoides* infection					
Uninfected	413 (49.2)	1			
Infected	426 (50.8)	1.26 (1.08-1.47)	0.003	1.18 (1.01-1.39)	0.040
*A.lumbricoides* infection intensity					
Uninfected	2115 (45.7)	1			
Low	279 (48.9)	1.15 (0.96-1.38)	0.451		
Medium	147 (54.9)	1.55 (1.20-2.01)			
Under weight	636 (48.7)	1.05 (0.92-1.19)	0.495		
Thin	276 (47.8)	1.07 (0.89-1.28)	0.455		
Stunted	94 (50.5)	1.16 (0.85-1.57)	0.345

**Table 3:** Results of Mantel-Haenszel adjusted odds ratios of the presence of hookworm infection on *Plasmodium falciparum* infection by sex, adjusting for age and *Ascaris lumbricoides* infection

Sex	Age (years)	% positive for hookworm	% positive for *Plasmodium*	Odds ratio (95 % CI), *χ* ^2^, *P* value^a^	Odds ratio (95 % CI), *χ* ^2^, *P* value
Boys					
	5-8	18.4 (115/624)	52.1 (325/624)	2.24 (1.43-3.50) 0.0003	
	9-10	16.4 (111/677)	51.7 (350/677)	1.21 (0.80-1.85) 0.354	1.40 (1.16-1.70) 8.85, P = 0.0004
	11-12	21.2 (154/728)	48.8 (355/728)	1.61 (1.12-2.33) 0.01	
	13-18	23.4 (176/753)	43.7 (329/753)	1.03 (0.73-1.45) 0.853	
			Homogeneity of ORs^b^: *χ* ^2^ = 8.42; 0.040		
Girls					
	5-8	12.5 (84/670)	47.2 (316/670)	1.77 (1.10-2.85) 0.02	
	9-10	14.3 (105/736)	47.0 (346/736)	1.51 (0.99-2.31) 0.05	
	11-12	13.6 (93/683)	42.3 (288/683)	0.97 (0.61-1.53) 0.896	1.33 (1.06-1.66) 3.41, P = 0.01
	13-18	34.3 (84/600)	38.7 (232/600)	1.19 (0.74-1.90 0.475	
			Homogeneity of ORs^b^: *χ* ^2^ = 3.84; 0.28		

^a^Age specific OR of *P. falciparum* in those concurrently infected with *A. lumbricoides* relative to those who are *A. lumbricoides* negative

^b^This test compares whether there was a significant difference between age-specific OR, hence whether the overall adjusted OR is valid

**Table 4:** Results of Mantel-Haenszel adjusted odds ratios of the presence of *Ascaris lumbricoides* infection on *Plasmodium falciparum* infection by sex, adjusting for age and hookworm infection

Sex	Age (years)	% positive for *A. lumbricoides*	% positive for *Plasmodium*	Odds ratio (95 % CI), *χ* ^2^ *P* value^a^	Odds ratio (95 % CI), χ ^2^ *P* value
Boys					
	5-8	17.9 (111/624)	52.1 (325/624)	0.99 (1.64-1.53) 0.97	
	9-10	13.6 (92/677)	51.7 (350/677)	1.42 (0.90-2.23) 0.131	1.12 (0.91-1.40) 1.13, P = 0.289
	11-12	14.2 (103/728)	48.8 (355/728)	1.03 (0.74-1.72) 0.581	
	13-18	12.9 (97/753)	43.7 (329/753)	1.03 (0.67-1.58) 0.898	
			Homogeneity of ORs^b^: *χ* ^2^ = 1.49; 0.685		
Girls					
	5-8	12.5 (137/670)	47.2 (316/670)	0.99 (0.67-1.45) 0.957	
	9-10	14.3 (125/736)	47.0 (346/736)	1.12 (0.76-1.65) 0.574	
	11-12	13.6 (98/683)	42.3 (288/683)	1.09 (0.70-1.70) 0.697	1.15 (0.93-1.42) 1.71, P = 0.192
	13-18	34.3 (76/600)	38.7 (232/600)	1.64 (1.00-2.67) 0.045	
			Homogeneity of ORs^b^: *χ* ^2^ = 2.70; 0.439		

^a^Age specific OR of *P. falciparum* in those concurrently infected with hookworm relative to those who are hookworm negative

^b^This test compares whether there was a significant difference between age-specific OR, hence whether the overall adjusted OR is valid

## Discussion

An increasing number of STH endemic countries in the world, including Kenya [15], are implementing school-based deworming programmes [21] and this study shows that only a quarter of school children are infected with STH species, whereas studies conducted a decade or more ago suggest that the majority of school-aged children living in western Kenya were infected with STH species [22, 23]. By contrast, malaria parasitaemia remains a common problem among Kenyan school children [16] and as a consequence, we found that 14.3 % of children harboured *STH-Plasmodium* coinfections, with hookworm-*Plasmodium* coinfection being the most common coinfection.

As expected, prevalence and density of *P. falciparum* infection varied by age group. However, patterns of STH infection showed little variation by age, owing perhaps to the relatively low levels of infection. Boys were more likely than girls to be infected with hookworm and *P. falciparum*, a possible reflection of behavior-related difference in exposure. Patterns of both single species infection and coinfection varied markedly by school and probably reflect a combination of behavioural and socioeconomic factors as well as small scale environmental factors [8, 18, 24, 25].

As shown in previous studies [1, 2, 5, 9, 26], we found an enduring association between hookworm and *P. falciparum*, after adjusting for age group, sex and presence of other helminth species (Table 4). Interestingly, in Mantel-Haenszel stratified analysis we found no such association between *A. lumbricoides* and *P. falciparum*, which is in contrast to previous studies conducted in Madagascar [27, 28] and Thailand [29], which found a negative effect of *A. lumbricoides* on *P. falciparum* infection, and a study in Mali which reported a positive association [30].

We recognize a number of limitations in our study. First, diagnosis was based on routine parasitological procedures and acknowledge that expert malaria microscopy may miss light infections when compared to more sensitive molecular methods [31] and that a single stool sample may underestimate the prevalence of helminth infection [32]. Second, the current survey did not collect household information and therefore is subject to potential confounding results. Detailed household-level information was collected only for those children who were found infected and therefore recruited to the main treatment trial (ClinicalTrials.gov NCT01658774), which prevented adjustment for potential confounding in the larger, cross-sectional data set.

## Conclusions

In summary, our study showed that helminth-*Plasmodium* coinfection is not uncommon in western Kenya, despite recent reductions in the prevalence of STH infection due to implementation of a national school-based deworming programme. Our study also corroborates previous studies that have demonstrated positive association between hookworm and *P. falciparum.* Further longitudinal studies are needed to investigate this association further, the clinical consequences of coinfection, and the impact of anthelminthic treatment on the risks of clinical malaria.

## Additional file

Additional file 1: Table S1. Description of study participants, with missing data vs. those not missing for 5980 children who provided a stool sample in 23 schools in Bumula District, western Kenya.

## References

[1] Salim N, Knopp S, Lweno O, Abdul U, Mohamed A, Schindler T, Rothen J, Masimba J, Kwaba D, Mohammed AS (2015). Distribution and Risk Factors for Plasmodium and Helminth Co-infections: A Cross-Sectional Survey among Children in Bagamoyo District, Coastal Region of Tanzania. PLoS Negl Trop Dis.

[2] Mazigo HD, Waihenya R, Lwambo NJ, Mnyone LL, Mahande AM, Seni J, Zinga M, Kapesa A, Kweka EJ, Mshana SE (2010). Co-infections with *Plasmodium falciparum* Schistosoma mansoni and intestinal helminths among schoolchildren in endemic areas of northwestern Tanzania. Parasit Vectors.

[3] Bisanzio D, Mutuku F, Bustinduy AL, Mungai PL, Muchiri EM, King CH, Kitron U (2014). Cross-sectional study of the burden of vector-borne and soil-transmitted polyparasitism in rural communities of Coast Province Kenya. PLoS Negl Trop Dis.

[4] Brooker SJ, Pullan RL, Gitonga CW, Ashton RA, Kolaczinski JH, Kabatereine NB, Snow RW (2012). Plasmodium-helminth coinfection and its sources of heterogeneity across East Africa. J Infect Dis.

[5] Kinung’hi SM, Magnussen P, Kaatano GM, Kishamawe C, Vennervald BJ (2014). Malaria and helminth co-infections in school and preschool children: a cross-sectional study in Magu district, north-western Tanzania. PLoS One.

[6] Mboera LE, Senkoro KP, Rumisha SF, Mayala BK, Shayo EH, Mlozi MR (2011). *Plasmodium falciparum* and helminth coinfections among schoolchildren in relation to agro-ecosystems in Mvomero District Tanzania. Acta Trop.

[7] Nkuo-Akenji TK, Chi PC, Cho JF, Ndamukong KK, Sumbele I (2006). Malaria and helminth co-infection in children living in a malaria endemic setting of mount Cameroon and predictors of anemia. J Parasitol.

[8] Pullan RL, Kabatereine NB, Bukirwa H, Staedke SG, Brooker S (2011). Heterogeneities and consequences of Plasmodium species and hookworm coinfection: a population based study in Uganda. J Infect Dis.

[9] Righetti AA, Glinz D, Adiossan LG, Koua AY, Niamke S, Hurrell RF, Wegmuller R, N’Goran EK, Utzinger J (2012). Interactions and potential implications of *Plasmodium falciparum*-hookworm coinfection in different age groups in south-central Cote d’Ivoire. PLoS Negl Trop Dis.

[10] Tshikuka JG, Scott ME, Gray-Donald K, Kalumba ON (1996). Multiple infection with Plasmodium and helminths in communities of low and relatively high socio-economic status. Ann Trop Med Parasit.

[11] Yapi RB, Hurlimann E, Houngbedji CA, Ndri PB, Silue KD, Soro G, Kouame FN, Vounatsou P, Furst T, N’Goran EK (2014). Infection and co-infection with helminths and Plasmodium among school children in Cote d’Ivoire: results from a National Cross-Sectional Survey. PLoS Negl Trop Dis.

[12] Maizels RM, Balic A, Gomez-Escobar N, Nair M, Taylor MD, Allen JE (2004). Helminth parasites–masters of regulation. Immunol Rev.

[13] Adegnika AA, Kremsner PG (2012). Epidemiology of malaria and helminth interaction: a review from 2001 to 2011. Curr Opin HIV AIDS.

[14] Brooker S, Akhwale W, Pullan R, Estambale B, Clarke SE, Snow RW, Hotez PJ (2007). Epidemiology of plasmodium-helminth co-infection in Africa: populations at risk, potential impact on anemia, and prospects for combining control. Am J Trop Med Hyg.

[15] Mwandawiro C, Nikolay B, Kihara JH, Ozier O, Mukoko DA, Mwanje MT, Hakobyan A, Pullan RL, Brooker SJ, Njenga SM (2013). Monitoring and evaluating the impact of national school-based deworming in Kenya: study design and baseline results. Parasit Vectors.

[16] Gitonga CW, Karanja PN, Kihara J, Mwanje M, Juma E, Snow RW, Noor AM, Brooker S (2010). Implementing school malaria surveys in Kenya: towards a national surveillance system. Malar J.

[17] Brooker S, Kabatereine NB, Smith JL, Mupfasoni D, Mwanje MT, Ndayishimiye O, Lwambo NJ, Mbotha D, Karanja P, Mwandawiro C (2009). An updated atlas of human helminth infections: the example of East Africa. Int J Health Geogr.

[18] Pullan RL, Gething PW, Smith JL, Mwandawiro CS, Sturrock HJ, Gitonga CW, Hay SI, Brooker S (2011). Spatial modelling of soil-transmitted helminth infections in Kenya: a disease control planning tool. PLoS Negl Trop Dis.

[19] WHO (2007). Anthroplus: growth reference 5–19 years.

[20] WHO (2002). WHO Expert Committe: Prevention and control of schistosomiasis and soil-transmitted helminthiasis. World Health Organ Tech Rep Ser.

[21] WHO (2012). Accelerating work to overcome the global impact of neglected tropical diseases – A roadmap for implementation.

[22] Handzel T, Karanja DM, Addiss DG, Hightower AW, Rosen DH, Colley DG, Andove J, Slutsker L, Secor WE (2003). Geographic distribution of schistosomiasis and soil-transmitted helminths in Western Kenya: implications for anthelminthic mass treatment. Am J Trop Med Hyg.

[23] Brooker S, Miguel EA, Moulin S, Luoba AI, Bundy DA, Kremer M (2000). Epidemiology of single and multiple species of helminth infections among school children in Busia District Kenya. East Afr Med J.

[24] Sang HC, Muchiri G, Ombok M, Odiere MR, Mwinzi PN (2014). Schistosoma haematobium hotspots in south Nyanza, western Kenya: prevalence, distribution and co-endemicity with Schistosoma mansoni and soil-transmitted helminths. Parasit Vectors.

[25] Odiere MR, Opisa S, Odhiambo G, Jura WG, Ayisi JM, Karanja DM, Mwinzi PN (2011). Geographical distribution of schistosomiasis and soil-transmitted helminths among school children in informal settlements in Kisumu City Western Kenya. J Parasitol.

[26] Humphries D, Mosites E, Otchere J, Twum WA, Woo L, Jones-Sanpei H, Harrison LM, Bungiro RD, Benham-Pyle B, Bimi L (2011). Epidemiology of hookworm infection in Kintampo North Municipality, Ghana: patterns of malaria coinfection, anemia, and albendazole treatment failure. Am J Trop Med Hyg.

[27] Brutus L, Watier L, Hanitrasoamampionona V, Razanatsoarilala H, Cot M (2007). Confirmation of the protective effect of *Ascaris lumbricoides* on *Plasmodium falciparum infection*: results of a randomized trial in Madagascar. Am J Trop Med Hyg.

[28] Brutus L, Watier L, Briand V, Hanitrasoamampionona V, Razanatsoarilala H, Cot M (2006). Parasitic co-infections: does *Ascaris lumbricoides* protect against *Plasmodium falciparum* infection?. Am J Trop Med Hyg.

[29] Nacher M (2011). Interactions between worms and malaria: good worms or bad worms?. Malar J.

[30] Le Hesran JY, Akiana J, el Ndiaye HM, Dia M, Senghor P, Konate L (2004). Severe malaria attack is associated with high prevalence of *Ascaris lumbricoides* infection among children in rural Senegal. Trans R Soc Trop Med Hyg.

[31] Banoo S, Bell D, Bossuyt P, Herring A, Mabey D, Poole F, Smith PG, Sriram N, Wongsrichanalai C, Linke R (2006). Evaluation of diagnostic tests for infectious diseases: general principles. Nat Rev Microbiol.

[32] Krauth SJ, Coulibaly JT, Knopp S, Traore M, N’Goran EK, Utzinger J (2012). An in-depth analysis of a piece of shit: distribution of *Schistosoma mansoni* and hookworm eggs in human stool. PLoS Negl Trop Dis.

