# Supplementary material for: Epidemiology of coinfection with soil transmitted helminths and Plasmodium falciparum among school children in Bumula District in western Kenya
Source: Parasit Vectors. 2015 Jun 11;8:314. doi: 10.1186/s13071-015-0891-5 (PMC4486705; doi:10.1186/s13071-015-0891-5)
Supplement: Additional file 1: Table S1. — Description of study participants, with missing data vs. those not missing for 5980 children who provided a stool sample in 23 schools in Bumula District, western Kenya. [file 13071_2015_891_MOESM1_ESM.docx]

**Table S1. Description of study participants, with missing data vs. those not missing for 5980 children who provided a stool sample in 23 schools in Bumula District, western Kenya.**

| **Characteristic; N (%) ^a^** |  | **Missing outcome data N=509** | **Outcome data available**  **N=5471** |
| --- | --- | --- | --- |
| Sex | Boys | 252 (49.6) | 2782 (50.9) |
|  | Girls | 256 (50.4) | 2689 (49.1) |
| Age (years) | Mean | 10.3 (2.3) | 10.5 (2.5) |
|  | 5-7 | 110 (24.5) | 1294 (23.7) |
|  | 8-10 | 116 (25.8) | 1413 (25.8 |
|  | 11-12 | 129 (28.7) | 1411 (25.8) |
|  | 13-18 | 94 (20.9) | 1353 (24.7) |
| Anthropometric status |  |  |  |
|  | % Underweight | 91 (17.8) | 1306 (23.9) |
|  | % Thin | 23 (4.5) | 577 (10.6) |
|  | % Stunted | 10 (2.0) | 186 (3.4) |
|  |  |  |  |
| *P. falciparum* | % Infected | 49 (59.8) | 2541 (46.4) |
| *P. falciparum* infection density^b^ |  | 1006 (468-2161) | 1921 (1518-2433) |
| Helminthinfection^c^ | Hookworm | 34 (6.7) [4.7-9.4] | 922 (16.9) [14.5-19.5] |
|  | *A. lumbricoides* | 44 (8.6) [5.6-13.1] | 839 (15.3) [12.2-19.2] |
|  | *T. trichiuura* | 1 (0.2) [0.02-1.5] | 21 (0.3) [0.02-0.7] |
|  | *S. mansoni* | 3 (0.6) [0.1-1.9] | 127 (2.3) [1.3-4.2] |
|  | Any STH | 70 (13.8) [9.9-19.0] | 1511 (27.6) [24.8-30.8] |
| Coinfection |  |  |  |
|  | Hookworm-*A. lumbricoides* | 9 (1.8) [0.6-4.8] | 259 (4.7) [3.7-6.1] |
|  | Hookworm-*P. falciparum* | 1 (1.2) [0.2-8.5] | 494 (9.0) [7.4-11.0] |
|  | *A.lumbricoides*-*P. falciparum* | 4 (4.9) [2.0-11.9] | 426 (7.8) [6.1-10.1] |
|  | Any STH-*P. falciparum* | 5 (6.1) [2.3-15.8] | 781 (14.3) [12.1-16.8] |
| Intensity of helminthInfection^d^ |  |  |  |
|  | Hookworm | 5 (3-5) | 41.2 (28-62) |
|  | *A. lumbridoides* | 617 (294-1297) | 758 (599- 1028) |

^a^Percentage of children is presented for categorised data. For continuous data mean (SD) is presented.

^b^Parasites per µl blood.

^c^Percentage of children infected [95% confidence intervals (CIs), taking into account clustering within schools]

^d^Mean infection intensity presented as eggs/gram faces (95% CIs).
